# Cross-sectional exercise-related differences in PTSD symptoms, psychological distress, physical pain, and sleep quality in trauma-exposed adults

**DOI:** 10.3389/fpsyg.2025.1445144

**Published:** 2025-06-19

**Authors:** Nicholas J. SantaBarbara, Erica R. Checko, Michelle M. Pebole, James W. Whitworth

**Affiliations:** ^1^School of Nursing and Health Sciences, Merrimack College, North Andover, MA, United States; ^2^Teachers College Columbia University, New York, NY, United States; ^3^National Center for PTSD, VA Boston Healthcare System, Boston, MA, United States; ^4^Department of Psychiatry, Boston University Chobanian & Avedisian School of Medicine, Boston, MA, United States; ^5^Translational Research Center for TBI and Stress Disorders, VA Boston Healthcare System, Boston, MA, United States

**Keywords:** posttraumatic stress disorder, physical activity, exercise, mental health, sleep, pain

## Abstract

**Purpose:**

Psychological trauma can lead to PTSD which is associated with numerous negative health outcomes. Exercise has beneficial effects on PTSD; however, the amount of exercise associated with these benefits remains unknown. To examine self-reported exercise-related differences in PTSD symptom severity, psychological distress, pain, and sleep quality in a national sample of trauma-exposed adults.

**Methods:**

Participants completed online assessments of exercise participation, PTSD symptom severity, psychological distress, pain, and sleep quality. Exercise level was defined as Active (≥24 on the Godin-Leisure Time Exercise Questionnaire [GLTEQ]), Insufficiently Active (1–23 on the GLTEQ), or Inactive (no reported exercise). MANCOVA was used to determine the relationship between exercise level (i.e., independent variable) and all outcomes (PTSD, distress, pain, sleep) with *post hoc* means comparison adjusted for age.

**Results:**

Participants’ (*n* = 500) mean age was 34.9 ± 13.0, and 68% were female. The overall model for exercise was significant, such that Active participants reported less PTSD symptom severity, psychological distress, and pain, and better sleep quality than Inactive participants.

**Conclusion:**

Meeting the recommended amount of weekly physical activity with moderate-to-vigorous exercise is associated with better physical and mental health among trauma survivors. Longitudinal research is needed to confirm these cross-sectional findings.

## 1 Introduction

Post-traumatic stress disorder (PTSD) is a mental health disorder that affects roughly 12 million US adults each year (National Institutes of Mental Health, n.d.). PTSD develops after a traumatic event that can include (but are not limited to) violent physical assaults, natural (or human) disasters (Milligan-Saville et al., 2018), accidents, cardiac events (Wilder Schaaf et al., 2013), partner- and sexual- violence (Dworkin et al., 2023), and/or military combat (Hughes et al., 2018). Although exposure to events like these are common (i.e., about one half of all U. S. adults will experience at least one traumatic event in their lives), not all trauma-exposed individuals develop PTSD (Hughes et al., 2018). However, trauma exposure can lead to disruptive subclinical PTSD symptoms, and when PTSD does occur it significantly impairs functioning and is associated with numerous negative health outcomes including psychological distress, physical pain, and poor sleep quality (Whitworth et al., 2017). In fact, individuals who develop PTSD are 80% more likely to develop other mental illnesses compared to those without PTSD (National Institutes of Mental Health, n.d.).

### 1.1 Psychological distress and PTSD

Regardless of the nature of the traumatic event, experiencing trauma can lead to psychological distress. For example, a 2013 systematic review found that following cardiac events, rates of depression ranged from 14 to 45% and anxiety from 13 to 61% (Wilder Schaaf et al., 2013). Observational findings from emergency service workers (i.e., police officer, fire fighter, paramedics) show that repeated trauma experience in the workplace can lead to significant and debilitating distress with rates as high at 10% in this study (Milligan-Saville et al., 2018). Further, another study analyzing data from 289,328 Iraq and Afghanistan veterans revealed that 50,432 (17.4%) received a clinical depression diagnosis with women experiencing a higher risk than men (Seal et al., 2009). Fortunately, a recent review highlights that among persons with differing traumatic experiences (e.g., first responders, natural disaster, fire fighters), the burden of distress can be attenuated through psychological interventions (Alshahrani et al., 2022). However, these types of interventions may be limited in accessibility and scalability. As such, strategies to reduce the burden of distress that are also highly scalable and sustainable are warranted.

### 1.2 Sleep quality and PTSD

Poor sleep quality (e.g., trouble falling and staying asleep, nightmares) is one of the hallmark symptoms of PTSD, is worse for those with co-occurring depressive symptoms (Weber et al., 2020), and results in more severe PTSD symptoms (Germain et al., 2004). Results from polysomnographic studies (i.e., a multimodal objective measure of sleep) among people with PTSD have observed rapid eye movement (REM) sleep attenuation and disruption (Baglioni et al., 2016) which is meaningful because REM sleep disturbances can threaten psychological resilience (Germain et al., 2004). Research exploring differences in diurnal rhythm between persons with PTSD compared to insomnia objectively demonstrates that persons with PTSD experience greater daily variability, stability and amplitude compared to persons with insomnia, with researchers suggesting one likely difference being unpredictable yet frequent nightmares (Mascaro et al., 2021). Still, poor sleep is a modifiable risk factor, and effective interventions that target sleep quality and potential nightmares as primary aims may expedite PTSD recovery.

### 1.3 Pain and PTSD

Pain is one of the most commonly reported physical symptoms of individuals with PTSD (Asmundson et al., 2002). Individuals who have pain from either a traumatic event (e.g., serious burns, physical assault) or morphological changes from a chronic disease (e.g., HIV) often report symptoms of PTSD (Asmundson et al., 2002). A documented range of 45–85% of patients who present for PTSD treatment also have a significant pain condition. The high co-prevalence of these conditions can lead to greater affective distress, interference in functioning and higher levels of disability than for those who live with either condition alone (Scioli et al., 2009). Thus, a better understanding of integrated treatment modalities that can complement existing evidence-based therapies for PTSD and/or pain to help manage and even mitigate such negative health consequences is needed.

### 1.4 Benefits of exercise for PTSD and related conditions

Exercise (i.e., purposeful physical activity done to improve health) has beneficial effects on PTSD and its negative health outcomes, including a reduction in trauma-related symptom severity and psychological distress. For example, observational, experimental, and qualitative evidence points to an inverse relationship between exercise and PTSD symptom severity (Whitworth and Ciccolo, 2016; Hegberg et al., 2019), with potential mechanisms that include exposure and desensitization to internal arousal cues (e.g., facilitating extinction), enhanced cognitive function, and reductions in inflammatory markers related to stress (Hegberg et al., 2019). Relationships between exercise, physical health conditions (e.g., pain) and other lifestyle-based behaviors (e.g., sleep) are understudied in trauma-exposed adults, reflecting the larger healthcare system in the United States which separates treatments for physical and mental conditions. However, several recent experimental trials demonstrate positive effects of exercise on PTSD symptoms and suggest benefits of exercise on sleep quality (Whitworth et al., 2019; Rosenbaum et al., 2015; Pebole et al., 2024). Increasing understanding of the relations among exercise, physical health and health promoting behaviors is relevant for creating integrated treatment approaches that incorporate exercise to improve functioning in physical and mental health domains.

However, the dose (i.e., amount) of exercise that is associated with a reduction in these negative outcomes is unclear. For instance, it is currently unknown if meeting the weekly physical activity guidelines set forth by the World Health Organization (i.e., 150 min/week or more of moderate-to-vigorous physical activity) (World Health Organization, 2018) with exercise is associated with physical and mental health among trauma-exposed adults. Understanding dose of exercise associated with better physical and mental health has significant implications for implementing exercise interventions and public health exercise promotion among communities exposed to trauma.

Thus, the purpose of this cross-sectional study was to examine exercise-related differences in PTSD symptom severity, psychological distress, pain, and sleep quality in a national sample of trauma-exposed adults. A more thorough understanding of the multifaceted links between these co-occurring symptoms and PTSD is timely in order to subsequently develop and deliver more targeted interventions. We hypothesized that participants who self-reported a weekly amount exercise consistent with meeting the recommended levels of weekly physical activity would report less PTSD symptom-severity, pain and psychological distress, and better sleep quality, relative to individuals who reported insufficient levels of weekly exercise or no weekly exercise.

## 2 Materials and methods

### 2.1 Participants and procedures

Baseline data from a larger longitudinal study examining exercise and PTSD were analyzed for this exploratory secondary analysis. The complete methods of the parent study can be referenced elsewhere (Whitworth et al., 2017). In brief, potential participants were recruited from major metropolitan areas across all four US regions (i.e., Northeast, South, Midwest, and West). Recruitment was accomplished through online classifieds, such as Craigslist. The classified listings provided a brief description of the study and a link to the study’s informed consent. Consenting individuals were then directed to an Internet platform and asked to complete a battery of questionnaires. To be eligible for the study, participants had to be living within the continental US, and have access to the Internet. All had to be at least 18 years in age, be able to read English, and report experiencing a traumatic event. However, disclosure of details relating to the trauma (e.g., trauma type, or when it occurred) were not required to participate. Individuals with additional histories of psychiatric illness other than PTSD (e.g., depression, anxiety, or substance use disorders) were not excluded from participation. Participants who did not meet these criteria based on an initial set of study questions were thanked for their participation but told they were ineligible at this time. Participants who completed the study were entered into a raffle to win a $50 gift card. The odds of winning were 1 in 25. The study was approved by the local Institutional Review Board at *blinded for review* and has been carried out in accordance with the Declaration of Helsinki. Data collection for this study between October 2015 and December 2016.

### 2.2 Measures

#### 2.2.1 Demographics questionnaire

Demographic data were self-reported and included age, gender, race/ethnicity, living condition, employment status, sexual orientation, education, income, veteran status, and height and weight.

#### 2.2.2 PTSD symptom severity

Post-traumatic stress disorder symptom severity was assessed with the PTSD Checklist-Civilian (PCL-C). The PCL-C is a 17-item, 5-point self-report scale used to assess PTSD symptom severity over the past month (Weathers et al., 1993). Items are ranked from “Not at all” to “Extremely” and correspond with the individual PTSD symptom clusters: re-experiencing, avoidance/numbing, and hyperarousal. Items are summed to represent a total PTSD symptom severity score. Valid total scores range from 17 to 85, with higher scores representing greater symptom severity. Scores of 30 or more are considered a positive screening for PTSD in the general US population. The PCL-C is a reliable and valid measure of PTSD symptom severity in numerous populations, including in the general public. It is strongly correlated with the Clinician Administered PTSD Scale (CAPS; *r* = 0.93) and is reliable when administered electronically (Campbell et al., 1999).

#### 2.2.3 Psychological distress

The Kessler Psychological Distress Scale (K10) is a self-report scale which assesses overall psychological distress over the past 4 weeks (Kessler et al., 2002). Each of the 10-items contains five points ranging from “Never” to “All of the time.” Valid scores range from 10 to 50, with higher scores representing greater levels of psychological distress. A score of 20 or more indicates a possible disorder causing psychological distress. The K10 is a valid and reliable measure of psychological distress (Kessler et al., 2002).

#### 2.2.4 Sleep quality

Global sleep quality for the past month was assessed with the full Pittsburgh Sleep Quality Index (PSQI). The PSQI is a self-report 19-item scale that assesses seven components of sleep, including sleep quality, latency, duration, efficiency, disturbances, use of sleep medications, and daytime dysfunction (Buysse et al., 1989). Scores range from 0 to 21. Higher scores represent worse global sleep quality and a cut-point of > 5 indicates poor global sleep quality. The PSQI is a reliable and valid assessment of global sleep quality (Buysse et al., 1989).

#### 2.2.5 Physical pain

Overall physical pain in the past month was assessed using the 2-item self-report Bodily Pain sub-scale of the Short Form Health Survey (Ware and Sherbourne, 1992). In this scale, the first item corresponds with pain intensity, consisting of 6-point ranking from “none” to “very severe.” The second item represents the amount pain has interfered with work or activities of daily living, and it has a 5-point ranking from “not at all” to “extremely.” Total scores range from 0 to 100 with lower scores representing more bodily pain. Scores less than 50 represent greater than average pain. The Pain sub-scale of the Short Form Health Survey is a simple, valid, and reliable measure of overall physical pain in the past month (Hawker et al., 2011).

#### 2.2.6 Leisure-time exercise

Self-reported exercise behaviors were measured using the Godin-Shephard Leisure-Time Exercise Questionnaire (GLTEQ) (Godin and Shephard, 1985). The GLTEQ asks individuals to report, “During a typical 7-Day period (a week), how many times on average do you do the following kinds of exercise for more than 15 min during your free time.” This was asked for strenuous (e.g., vigorous running or cycling), moderate (e.g., non-exhaustive sports, jogging, or weight training), and minimal intensity exercise (e.g., yoga, or easy walking). The GLTEQ is scored by multiplying the frequency of strenuous, moderate, and minimal intensity exercise by corresponding metabolic equivalent values 9, 5, and 3, respectively. For interpretation, individuals who achieved a score of ≥24 from either moderate, or strenuous intensity exercise, or a combination of both (i.e., total leisure-time exercise) were classified as “Active” and were likely to have met the weekly physical activity recommendations of ≥150 min/week of moderate-to-vigorous intensity physical activity (Amireault and Godin, 2015). Individuals who scored > 0 but < 24 were considered “Insufficiently Active” (i.e., reported some weekly leisure-time exercise, but below the recommended levels). Individuals who reported no weekly exercise (i.e., a score of 0) were categorized as “Inactive.” The GLTEQ is a reliable and valid measure of total leisure-time exercise, as well as strenuous, moderate, and minimal intensity exercise (Amireault and Godin, 2015).

### 2.3 Data analysis

Descriptive statistics were used to describe the aggregate sample and are presented as mean (standard deviation) for continuous variables and n (percentage) for categorical variables. As a preliminary step, associations between potential confounders, (e.g., demographics) and predictor (i.e., exercise) and outcome (i.e., PTSD symptom severity, psychological distress, pain, sleep quality) were considered. Pearson’s *r* was used for continuous variables and chi-square tests were used for categorical variables. Any variable significantly associated with both outcome and predictor were considered confounders and adjusted for in the subsequent analyses.

Multivariate analysis of variance was then conducted to determine the overall relationship between exercise participation (i.e., independent variable) and PTSD symptom severity, pain, psychological distress, and sleep quality (i.e., dependent variables). In our preliminary step, we found that age was a significant confound, thus, a multivariate analysis of covariance was conducted adjusting for age.

Lastly, pairwise comparisons of exercise level (i.e., Active = GLTEQ score ≥24; Insufficiently Active = GLTEQ score of 1–23; and Inactive = GLTEQ score of 0) for each dependent variable were conducted with Bonferroni correction. The threshold for statistical significance was *p* < 0.050.

## 3 Results

A total of 741 individuals enrolled in the study. Five hundred participants completed the baseline electronic survey and were included in the final analyses. Of the 239 individuals excluded from the study, 107 consented to participate, but did not respond to any question (i.e., there is no descriptive data on them). A comparison of the remaining 132 individuals excluded from the final analysis to those who were included, found they were significantly more likely to identify as male (*p* < 0.05). However, there were no other observed significant differences between those included and excluded. See Table 1 for a complete description of the sample characteristics, and Table 2 for a summary of PTSD symptom severity, pain, psychological distress, and sleep quality organized by activity level. In brief, participants’ mean age was 34.9 (13.0) years and body mass index was 27.8 (7.5). Most participants identified as female (68.8%), White (69.2%), heterosexual (73.6%), and were non-Latino/a (84.4%). Almost half (48.4%) of the participants were considered Active, 25.6% and Insufficiently Active, and 26.0% Inactive. The mean PCL-C score was 55.7 (16.4), K10 30.6 (9.7), physical pain 61.7 (27.7), and PSQI 10.6 (4.5).

**Table 1:** Sample characteristics (*n* = 500).

Characteristic	*n* (%)
Gender	
Male	134 (26.8)
Female	344 (68.8)
Transgender	16 (3.2)
Other	6 (1.2)
Race	
American Indian/Alaskan Native	14 (10.4)
Asian/ Native Hawaiian or Pacific Islander	42 (8.4)
Black or African American	42 (8.4)
White	346 (69.2)
Other/Do not know	56 (11.2)
Latino/a	78 (15.6)
Education	
High school or less	70 (14.0)
Some college/vocational school	135 (27.0)
Completed college/vocational school	295 (59.0)
Employment status	
Full-time	167 (33.4)
Part-time	90 (18.0)
Unemployed	112 (22.4)
Student	61 (12.2)
Other	70 (14.0)
Household income	
$25,000 or less	196 (39.2)
$25,001–$60,000	141 (28.2)
$60,001–$100,000	87 (17.4)
$100,001 or more	41 (8.2)
Do not know	35 (7.0)
Military veteran (Yes)	65 (13.0)
Sexual orientation	
Heterosexual	368 (73.6)
Homosexual	40 (8.0)
Bisexual	67 (13.4)
Other	25 (5.0)
Physical activity status	
Inactive	130 (26.0)
Insufficiently Active	128 (25.6)
Active	242 (48.4)
Psychiatric history	
PTSD	311 (62.2)
Anxiety disorder	281 (56.2)
Depression	316 (63.2)
Schizophrenia	16 (3.2)
Bipolar disorder	75 (15.0)
Alcohol use disorder	30 (6.0)
Substance use disorder	32 (6.4)

	Mean (standard deviation)
Age	34.9 (13.0)
Body mass index (*n* = 316)	27.8 (7.5)
PCL-C	55.7 (16.4)
K10	30.6 (9.7)
Chronic pain	61.7 (27.7)
PSQI	10.6 (4.5)

PCL-C = PTSD Checklist Civilian; K10 = Kessler Psychological Distress Scale; PSQI = Pittsburgh Sleep Quality Index. Height and weight data were collected on 316 participants, so body mass index could not be calculated for the entire sample.

**Table 2:** Group-level summary statistics for PTSD symptoms, psychological distress, chronic pain, and sleep.

Activity status	PTSD symptoms	Psychological distress	Chronic pain	Sleep quality	
	Mean (SD)	Mean (SD)	Mean (SD)	Mean (SD)	n
Inactive	59.9 (15.7)	32.6 (9.3)	54.5 (28.3)	12.2 (4.7)	130
Insufficiently Active	55.4 (15.0)	31.1 (9.0)	61.8 (25.2)	10.2 (4.1)	128
Active	53.6 (17.2)	29.3 (10.1)	65.5 (28.0)	9.9 (4.5)	242

SD = standard deviation; total *n* = 500.

The assumptions of equality of covariance matrices and error variances were evaluated with Box’s M (*p* > 0.05) and Levene’s Test (all *p’s*, > 0.05), respectively. The overall multivariate model for exercise was significant, *F*(8, 986) = 3.71, *p* < 0.001; Wilk’s *λ* = 0.942. Results for the between-subjects tests were significant for dependent variables: PTSD symptom severity [*F*(2, 496) = 7.98, *p* < 0.001], psychological distress [*F*(2, 496) = 7.54, *p* = 0.001], physical pain [*F*(2, 496) = 4.79, *p* = 0.009], and sleep quality [*F*(2, 496) = 11.89, *p* < 0.001]. Age was a significant covariate for PTSD symptom severity, psychological stress, and physical pain (*p*’s < 0.050), but not sleep quality (*p* = 0.329). Complete results for the pairwise comparisons can be found in Table 3. In summary, individuals who were Active, reported significantly less PTSD symptom severity, psychological distress, pain, and better sleep quality, relative to Inactive participants (*p*’s < 0.010). Additionally, individuals who were Insufficiently Active reported significantly less PTSD symptom severity and better sleep quality than Inactive participants (*p*’s < 0.050). Insufficiently Active and Active individuals did not differ significantly for any dependent variable (*p*’s > 0.050).

**Table 3:** Pairwise comparison between PTSD, distress, pain, and sleep, by physical activity status.

Dependent variable	PA level comparison	Mean difference (95% CI)	SE	*p*-value
PTSD symptoms	Inactive vs. Insufficiently active	5.29 (0.38, 10.19)	2.04	0.030*
	Inactive vs. Active	7.11 (2.82, 11.41)	1.79	<0.001**
	Insufficiently active vs. Active	1.83 (−2.41, 6.08)	1.77	0.904
Psychological distress	Inactive vs. Insufficiently Active	2.24 (−0.64, 5.11)	1.20	0.188
	Inactive vs. Active	4.05 (1.53, 6.57)	1.05	<0.001**
	Insufficiently Active vs. Active	1.81 (−0.69, 4.30)	1.04	0.246
Chronic pain	Inactive vs. Insufficiently Active	−5.45 (−13.68, 2.77)	3.42	0.336
	Inactive vs. Active	−9.25 (−16.44, −2.05)	3.00	0.006**
	Insufficiently Active vs. Active	−3.80 (−10.92, 3.33)	2.97	0.604
Sleep quality	Inactive vs. Insufficiently Active	2.08 (0.73, 3.43)	0.53	0.001**
	Inactive vs. Active	2.33 (1.15, 3.51)	0.49	<0.001**
	Insufficiently Active vs. Active	0.25 (−0.92, 1.42)	0.49	0.999

PTSD = posttraumatic stress disorder; PA = physical activity; CI = confidence interval; SE = standard error. * and ** Denote statistical significance at *p* < 0.050 and *p* < 0.010, respectively. *P*-values were adjusted for multiple comparisons, using Bonferroni correction factor.

## 4 Discussion

The purpose of this online observational study was to explore relationships between level of self-reported exercise (i.e., Inactive, Insufficiently Active, and Active), PTSD symptom severity, psychological distress, physical pain, and sleep quality. We hypothesized that a greater amount of exercise would be associated with lower PTSD symptom severity, less psychological distress, less pain, and better sleep quality among trauma-exposed individuals. Indeed, the results showed that Active participants reported significantly lower PTSD symptoms, psychological distress, less pain, and better sleep quality than those who reported being Inactive. While cross-sectional, these results may support the importance of trauma-exposed individuals meeting the recommended amount of weekly physical activity through exercise (i.e., consistent with a score ≥24 on the GLTEQ, or ≥150 min per week of moderate-to-vigorous intensity exercise). Another key finding of this study is Insufficiently Active individuals also reported significantly lower PTSD symptoms and better sleep quality than Inactive participants, which provides preliminary support for a dose–response relationship between exercise, PTSD symptoms and sleep quality.

These findings largely support the results of research in related areas. For instance, a recent large study of exercise dose and generalized anxiety disorder (GAD) from The Irish Longitudinal Study on Ageing (TILDA) reported a dose–response relationship between moderate-to-vigorous exercise participation and the risk for GAD (Herring et al., 2024). Importantly, the largest reported decrease in risk for GAD was seen among individuals transitioning from inactive (i.e., no reported exercise) to insufficiently active (i.e., some exercise, but less than recommended amounts). Similar results for physical activity and depression were published in a recent meta-analysis (Pearce et al., 2022). Specially, a dose–response effect of physical activity on risk for depression, with the largest observed benefit going from inactive to insufficiently active. This mirrors our findings for PTSD symptom severity and sleep quality (see Table 3). In contrast, we did not observe significant differences in bodily pain and psychological distress between the Inactive and Insufficiently Active groups, or between the Insufficiently Active and Active groups. However, Active individuals did report significantly less psychological distress and bodily pain than Inactive individuals. These results are largely consistent with the field, suggesting that exercise has a beneficial effect on both pain (Geneen et al., 2017) and psychological distress (Singh et al., 2023).

An important characteristic of this study is that multifaceted approach to evaluating associations between exercise and physical and mental health, rather than evaluated PTSD, psychological distress, pain, and sleep quality in isolation. This is critical, as there are high rates of comorbidity among individuals with psychiatric disorders, insomnia and pain. This is clearly demonstrated in our sample where on average PTSD symptom severity, psychological distress and pain were elevated, and sleep quality was poor.

The association of exercise and multiple components of mental and physical health has potentially broad implications for exercise as a tool in the treatment and prevention of PTSD and related conditions. Stress and coping theory help provide a useful framework these relationships (Folkman and Moskowitz, 2004). Specifically, it is possible that exercise may be a useful pluripotent coping strategy for clinicians to promote physical and mental wellness for trauma survivors. Exercise may also be an opportunity for trauma survivors have an element of control and autonomy in their mental and physical health journey (e.g., choosing what types of exercise to do, where, when, and with whom (Sheppard-Perkins et al., 2022; Pebole et al., 2021)). As some studies suggest, regular exercise participation may reduce PTSD symptom severity, distress, lessen pain, and improve global sleep quality (Hegberg et al., 2019; Whitworth et al., 2019; Rosenbaum et al., 2015). However, it is equally plausible that the combined influence of poor mental health, insomnia, and physical pain, prevent regular exercise to a greater extent than each condition independently (Morasco et al., 2013; Talbot et al., 2014). In fact, some research supports this, suggesting that individuals affected by these conditions, or having greater symptom severity of these conditions, are less likely to exercise (Hall et al., 2015; Hall et al., 2020). For example, individual with severe PTSD may be more likely to rely on avoidance or numbing based coping strategies (e.g., avoiding busy public spaces, like gyms or parks, and/or isolation/distancing). Perhaps the most likely scenario is a combination of both cases, such that both exercise and an individual’s current health simultaneously exert their influence in a bidirectional game of tug-of-war. It is therefore recommended that researchers who are interested in developing exercise interventions for trauma-exposed adults consider this bi-directional relationship and offer support, particularly for those who are sedentary, to engage these individuals more effectively in exercise.

There were several strengths and limitations to this study. First, we recruited a national sample of trauma-exposed adults. This increases the external validity of our findings to a greater extent than data from treatment-seeking adults from a hospital setting. Secondly, the GLTEQ is a validated assessment of exercise that can be a reliable way to gauge whether participants are meeting the recommended weekly amount of physical activity set forth by the World Health Organization. Further, the GLTEQ helps to extend findings from previous exercise and PTSD research which have focused heavily on single-item measures of exercise (Hegberg et al., 2019). Despite these strengths, this study has limitations. First, the data used in this study is cross-sectional, thus the directionality of the observed relationships cannot be determined from this study alone. Further, this was a secondary analysis, and the results should be considered exploratory and not confirmatory. The observational and online study design also presents some challenges. For example, despite sampling from all regions of the United States, the data is not nationally representative, and certain demographics are likely over-represented (e.g., non-Hispanic White women), while others are likely under-represented (e.g., Black men). Indeed, analysis of the individuals who did not complete the study, suggests they were more likely to identify as male. Other factors, such as the low compensation for participation (i.e., a gift card raffle) may have deterred some individuals from participating. As such, these data may not generalize to all peoples, especially harder to reach populations, and those without an internet connection (e.g., some rural communities and individuals without stable housing). The self-report nature of this study may also subject the data to recall biases and other inaccurate reporting associated with self-report data collection (Haskell, 2012). Further, the measurement of pain in this study is limited to pain in the past month and cannot determine if the reported physical pain was chronic (i.e., lasting more than 12 weeks). As previously described, pain commonly co-occurs with PTSD and is known to further interfere with functioning. As such, more comprehensive assessment of pain is recommended in future physical activity and PTSD research.

## 5 Conclusion

Greater amount of self-reported exercise is cross-sectionally associated with lower PTSD symptom severity, less psychological distress, physical pain, and better sleep quality among trauma-exposed adults. Meeting the recommended amount of weekly physical activity with moderate-to-vigorous exercise is associated with the largest differences. However, achieving some weekly exercise but not meeting the recommended amount (i.e., Insufficiently Active) was also associated lower PTSD symptom severity and better sleep quality than no physical activity.

Practical Implications and Future Directions: The results of this study suggest even small amounts of exercise (i.e., less than the recommended weekly dose of exercise) may be beneficial for trauma-exposed individuals – something is better than nothing. This is encouraging, and supports individuals becoming more active gradually, over time, at their own pace.

Future studies are needed to examine the longitudinal course of exercise participation among trauma-exposed individuals to determine its potential role in the treatment and prevention of PTSD, and related conditions. This work would be further enhanced by more detailed investigations of the parameters of exercise, For instance, comparisons between exercise mode (e.g., aerobic and resistance exercise), as well as patient/trauma survivor exercise preferences.

## Data Availability

The dataset presented in this article is not readily available because of security protocols and privacy regulations, but they may be made available on reasonable request by the VA Boston Healthcare System or by contacting the corresponding author to facilitate the request.
